# Initial Experience of the Feasibility of Single-Incision Laparoscopic Appendectomy in Different Clinical Conditions

**DOI:** 10.1155/2010/240260

**Published:** 2010-04-13

**Authors:** Jyrki Kössi, Markku Luostarinen

**Affiliations:** Department of Surgery, Päijät-Häme Central Hospital, Keskussairaalankatu 7, 15850 Lahti, Finland

## Abstract

*Introduction*. Single-incision laparoscopic surgery (SILS) is a new technique developed for performing operations without a visible scar. Preliminary studies have reported the use of the technique mainly in cholecystectomy and appendectomy. We evaluated the feasibility of the technique in various appendicitis conditions including children, fertile women and obese patients. *Materials and Methods*. SILS technique was used in a random sample of patients hospitalised for suspected appendicitis. The ordinary diagnostic laparoscopy was performed and the appendix was removed if needed. The ligation of appendix was performed by thread loop, absorbable clip or endoscopic stapler. The details regarding the recovery of patients were collected prospectively. *Results*. Ten SILS procedures were performed without conversions or complications. The patient series included uncomplicated and complicated appendicitis patients. The mean age of the patients was 37 years (range 13–63), mean BMI was 26 (range 18–31), mean operative time was 40 minutes (range 18–31), and mean postoperative stay was 2 days (range 1–5). *Conclusions*. SILS technique is feasible for obese patients, uncomplicated and complicated appendicitis as well as for exploratory laparoscopy. Most common methods to ligate appendix are feasible with SILS technique. The true benefit of the technique should be assessed by randomised controlled trials.

## 1. Introduction

During the era of laparoscopic surgery common trend has been towards less invasive techniques and a natural extension of the trend is to perform operations without scars. The most prominent techniques representing scarless surgery are transumbilical single-incision laparoscopic surgery (SILS) and natural orifice transluminal endoscopic surgery (NOTES). As the latter is still struggling with some technical and equipmental difficulties, SILS seems to be more ready for wider use in surgical community. There are reliable and simple equipment available for SILS procedures, and the operative technique, although different than in conventional laparoscopy, is probably easier to learn compared to NOTES technique. 

Several operations have, thus, been until now performed by SILS technique including, for example, cholecystectomy, appendectomy, splenectomy, and sleeve gastrectomy. The most abundant are publications presenting results of SILS cholecystectomy [1–4] and results obtained in pediatric surgery [5–7]. All these reports have indicated that the SILS technique is safe and feasible in these surgical populations and that the operative time with this new technique is reasonable.

Appendectomy is the most common abdominal operation performed as an emergency basis in the western world [8]. The advantage of laparoscopic technique over the conventional technique has been proven especially in fertile women and obese patients [9–11]. SILS appendectomy may be even more advantageous to the patients by eliminating the scars and potentially diminishing postoperative pain. However, the role of the SILS appendectomy is still evolving since all published reports of the technique should be regarded as preliminary [5–7, 12]. More studies evaluating the technique in different clinical situations as well as randomised controlled trials are needed in order to assess the real benefits of the SILS appendectomy in general surgical practice.

The aim of the present study was to evaluate the feasibility of SILS diagnostic laparoscopy and appendectomy in heterogenic patient population presenting with symptoms suggestive for appendicitis. The suitability of different equipments for appendiceal ligation was also evaluated as well as the learning of the procedure.

## 2. Materials and Methods

This report is a case series of 10 patients admitted to Päijät-Häme Central Hospital due to right lower abdominal pain suggestive for appendicitis. All patients were clinically deemed to have high suspicion of appendicitis and were scheduled for emergency single-incision laparoscopy and subsequent appendectomy, if needed. The intention was to recruit a heterogeneous patient population to the procedure including, for example, children, fertile women, and obese patients. The operation was performed transumbilically using SILS port (Covidien, Norwalk, CT, USA). Firstly, intraumbilical cutaneous vertical incision was made and the umbilicus was detached from the fascia. The fascia was opened (2-3 cm) and the SILS port was introduced into the abdomen. After that, three 5 mm trocars were put through the port and the pneumoperitoneum was induced. A 5 mm 30-degree optic was used in all operations. One straight and one curved grasper (Roticulated endo grasp, Auto Suture, Norwalk, CT, USA) were introduced into the abdomen and right lower abdominal quadrant was explored and the operation was continued according to the findings. When deemed necessary, appendix was removed. In all patients mesoappendix was dealt with bipolar electrocautery and laparoscopic scissors. If extensive dissection was needed, dissecting monopolar hook was additionally used. The ligation of appendix was performed by thread loop (Endoloop, Ethicon, Somerville, NJ, USA), absorbable clip (Lapro-clip, Auto Suture, Norwalk, CT, USA), or endoscopic stapler (Endogia, Auto Suture, Norwalk, CT, USA). When clip or endoscopic stapler was used, one of the 5 mm ports was replaced by 12 mm port (Versastep, Auto Suture, Norwalk, CT, USA). The appendix was extracted with a pouch (Endocatch Gold, Auto Suture, Norwalk, CT, USA). If the appendix proved to be normal, standard diagnostic laparoscopy was performed including the examination of 100 cm of distal ileus, female genital organs, ascending colon, sigmoid colon, and gallbladder. At the end of the procedure, fascia was closed with continuous absorbable suture, umbilicus was refixed to the fascia, and the skin was closed with absorbable sutures. After discharge details of intraoperative and postoperative data were recorded.

## 3. Results

Altogether 10 patients were operated on by the SILS technique. There were 5 men and 5 women. Nine patients had appendectomy while one patient with sigmoid diverticulitis had only diagnostic laparoscopy. The mean age of the patients was 37 years (range 13–63), mean BMI was 26 (range 18–31), and the mean operative time was 40 minutes (range 23–50). The mean postoperative stay was 2 days (range 1–5). There were no conversions, no wound complications, or other complications among patients. The operative finding, operative time, and some other clinical details of different patients are shown in Table 1. All types of appendicitis from uncomplicated disease to disease with diffuse peritonitis were represented in our patient series. The patient with perforated appendicitis and diffuse peritonitis made an uneventful recovery although she spent 5 days in the hospital due to the therapy for diffuse peritonitis. Another patient with local dense inflammatory reaction and incipient abscessus formation could be operated by SILS technique and recovered normally. The method was also suitable for the most obese patient in our series. In the young female patient with rupture of ovarian cyst the exploratory laparoscopy with therapeutic intervention could be performed without difficulties by SILS technique.

## 4. Discussion

Appendectomy is the most common abdominal emergency operation in the western world. More and more appendectomies are currently performed laparoscopically due to the fact that the technique offers advantages to patients in terms of more accurate diagnosis, diminished wound infections, and more rapid recovery [9]. Compared to traditional laparoscopy, SILS appendectomy results surely in better cosmesis but additional benefits, for example, in terms of more rapid recovery have not been proven scientifically. However, randomised controlled clinical trials are urgently needed to define the role of SILS appendectomy in the modern surgical armamentarium.

Always when a new technique is introduced to the surgical community, the focus should be concentrated on the feasibility, safety, and clinical advantage of the method. Further, safety is highly dependent on how easily the new technique can be learned by average surgeons. It is well acknowledged that the implementation phase of new techniques is associated with an increased risk of complications emphasizing the importance of thorough training and education. The SILS technique differs from traditional laparoscopic technique remarkably by the use of the grasping and dissecting instruments. Due to the vicinity of the ports at the fascial plane, the operative technique necessitates crossing of the instruments (or specially designed instruments) making the procedure more challenging and initiating new learning curve for surgeon. Thus, transition from conventional laparoscopy to SILS is demanding, initiates new learning curve for surgeons, and increases initial operative time as shown in a previous study [12]. The most common conventional laparoscopic technique for appendectomy uses three ports meaning that the removal of appendix by SILS technique is performed principally similarly compared to traditional laparoscopy. Secondly, appendectomy is relatively easy operation performed in a relatively safe abdominal area decreasing the risk of disastrous complications that may happen, for example, in cholecystectomy. Further, SILS appendectomy can be performed properly by one straight instrument and one curved instrument making the procedure easier compared to use of two curved instruments.

When performing appendectomy, one must be prepared for different abdominal findings. The appendicitis may be oedematic, gangrenous, perforated with varying degree of peritonitis, or even associated with peritoneal abscess. The technique chosen to treat the patients should be suitable for all these clinical situations. In the present patient series there were both uncomplicated and complicated cases with even different degrees of peritonitis. All our patients could be operated by SILS technique without conversions or additional ports and they had an uneventful recovery. Further, the mean operating time was 40 minutes comparing well to the operating time of conventional laparoscopic appendectomy in our hospital (mean 43 minutes, range 18–103) and in a recent Cochrane review (mean 23.5–102 minutes) [9]. According to our experience, although limited, SILS technique seems to be suitable for variety of appendiceal infections.

Another issue is the feasibility of SILS technique for performing exploratory laparoscopy when surgeon encounters a normal appendix and the nature of the disease should be determined. According to our experience a proper diagnostic laparoscopy can be performed by SILS technique relatively easily and rapidly. The examination of distal ileum, female genital organs, and other organs situated in pelvic area could be accomplished without difficulties. 

We tried intentionally different techniques for ligation of appendix in order to find out how feasible they are. Probably the most common methods to ligate appendiceal stump are thread loop, absorbable clip, and endoscopic stapler. All these options seemed to be suitable for SILS appendectomy. However, the easiest and fastest method in our hands was endoscopic stapler that has been suggested to lower the risk of postoperative intraabdominal surgical-site infection and the need for readmission to hospital [13], although a recent systematic review did not support this view [14].

According to literature especially obese patients benefit of laparoscopic appendectomy compared to open one and laparoscopy should be preferred technique for these patients [9–11]. It is, thus, important that new mini-invasive operative techniques are suitable for this patient population too. As shown in Table 1 a male patient with BMI 31 could be operated on by SILS technique in a reasonable time and his postoperative recovery was excellent. Although our experience with the technique is limited, it can be suggested that SILS technique for appendectomy is probably as suitable as traditional technique in obese patients.

Few of our patients were adolescent females who may be very aware of their body image. Abdominal scar may have influence on their quality of life and they may appreciate that their operations have been performed without a visible scar. However, to our knowledge there is only one small study in the literature focusing on the issue of the influence of abdominal scar on the cosmesis and body image showing no difference between open and traditional laparoscopic appendectomies [15]. As the main advantage of the SILS technique is that the visible scar can be avoided, further studies evaluating the issue urgently needed. Conventional laparoscopic appendectomy produces relatively small scars and the superiority of SILS in that respect remains to be shown. Further, the importance of abdominal scar may be age related since a limited survey among scrub nurses in our hospital revealed that young nurses would have scarless operation if it were available, but older ones did not see the issue so important.

Although SILS technique looks promising and offers some potential benefits for patients compared to conventional laparoscopy, two possible disadvantages should be considered. SILS technique may be associated with increased risk of hernias. The technique necessitates fascial incision through the abdominal midline that has been considered to be prone to hernia formation. Further, the fascial incision is more traumatic compared to 5 or 12 mm trocar wounds made with dilating trocars. The second possible disadvantage is the additional costs caused by the procedure-specific port and instruments. These extra operative costs should be taken into account in the current trend towards costeffectiveness in healthcare.

## 5. Conclusions

SILS technique is feasible for a variety of appendiceal inflammatory conditions and for explorative laparoscopy. The technique suits well for obese patients and different technical methods for appendiceal ligation can be easily used. Appendectomy is suitable procedure for the training of SILS technique. The technique may have few disadvantages and the true benefit of the technique remains to be shown by randomised controlled trials. 

## Figures and Tables

**Table 1:** 

Patient description	Operative finding	Operation	Operative time (min)	Discharge (days)	Note
Male, 40 years	Appendicitis	Appendectomy	38	1	Typical uncomplicated appendicitis
Female, 18 years	Perforated appendicitis, covered by terminal ileum	Appendectomy	44	4	Restricted infection, incipient abscessus formation
Female, 63 years	Perforated appendicitis, diffuse peritonitis	Appendectomy, lavation	50	5	Hospital stay prolonged due to peritonitis
Male, 31 years	Appendicitis	Appendectomy	37	1	Obese patient, BMI 31, operative time reasonable
Female, 16 years	Ovarian cyst rupture	Appendectomy, explorative laparoscopy	34	2	Aspiration of pelvic fluid collection
